# Severe delayed gastric emptying combined with enteroparalysis after pancreaticoduodenectomy: a case report

**DOI:** 10.3389/fsurg.2026.1938654

**Published:** 2026-10-22

**Authors:** Zhili Wan, Dingquan Wu, Yuhang Yang, Guizhong Li, Bin Liu, Yongfan Chen, Liangbi Zhou

**Affiliations:** 1 Hepatic-biliary Pancreatic Department, Chongqing Hospital of The First Affiliated Hospital of Guangzhou University of Chinese Medicine (Chongqing Beibei Hospital of Traditional Chinese Medicine), Chongqing, China; 2 General Surgery Department, Chongqing Hospital of The First Affiliated Hospital of Guangzhou University of Chinese Medicine (Chongqing Beibei Hospital of Traditional Chinese Medicine), Chongqing, China

**Keywords:** delayed gastric emptying, enteroparalysis, gastrointestinal motility disorder, nutritional support, pancreaticoduodenectomy

## Abstract

**Background:**

Pancreaticoduodenectomy is an important surgical procedure for treating diseases of the pancreatic head, periampullary region, and related conditions. However, due to its extensive surgical scope, long duration, and involvement of complex digestive tract reconstruction, postoperative gastrointestinal dysfunction remains relatively common. Among these, delayed gastric emptying (DGE) is a common complication after pancreaticoduodenectomy, while the occurrence of severe DGE combined with enteroparalysis is relatively rare, and its diagnosis and treatment remain challenging.

**Case presentation:**

This article reports the case of a 69-year-old female patient who developed severe DGE complicated by intestinal paralysis after pancreaticoduodenectomy. The patient persistently experienced nausea, vomiting, and the inability to tolerate oral intake postoperatively. Although imaging studies suggested restricted passage at the gastrointestinal anastomosis, raising suspicion of obstruction, further laparoscopic exploration and gastroscopy revealed no definite mechanical obstruction. The patient received gastrointestinal decompression, nutritional support, internal environment management, and rehabilitation therapy. Enteral nutritional support was established through a jejunal feeding tube. After long-term comprehensive treatment, the patient's gastrointestinal function gradually recovered and eventually returned to oral intake. However, long-term follow-up showed that despite the recovery of gastrointestinal function, the patient still had a certain degree of decline in physical function and limited ability in activities of daily living.

**Conclusion:**

Severe delayed gastric emptying complicated by enteroparalysis after pancreaticoduodenectomy is a rare but clinically challenging gastrointestinal dysfunction. This case suggests that persistent postoperative gastrointestinal dysfunction may present with clinical features resembling mechanical obstruction, therefore requiring comprehensive assessment integrating imaging, endoscopy, and dynamic clinical changes.

## Introduction

Pancreaticoduodenectomy (PD) is an important surgical approach for treating diseases related to the pancreatic head, periampullary region, bile duct, and duodenum (1). Due to the complex anatomical structures of the pancreatic head and duodenal region, and because the surgery involves multiple digestive tract reconstruction procedures, PD remains one of the more technically demanding operations in abdominal surgery. Although surgical techniques, perioperative management, and postoperative monitoring have continuously improved in recent years, a variety of complications may still occur after PD, including pancreatic fistula, hemorrhage, delayed gastric emptying (DGE), and other gastrointestinal dysfunctions (2).

DGE is one of the common gastrointestinal complications after PD (3, 4), and the International Study Group of Pancreatic Surgery (ISGPS) has proposed the relevant definition and grading criteria (Table 1) (5). The main characteristic of DGE is that, in the absence of mechanical obstruction, patients are unable to return to normal oral intake after surgery, possibly leading to deterioration of nutritional status, prolonged hospital stay, and impaired postoperative recovery (6). Most patients with DGE can gradually recover after gastrointestinal decompression, nutritional support, and other conservative treatments, but some patients may develop persistent gastrointestinal motility disorders (7). Postoperative enteroparalysis is a functional disease characterized by prolonged recovery of gastrointestinal function after surgery due to non-mechanical factors (8). Its main manifestations include nausea, vomiting, abdomen distension, delayed passage of flatus and stool, and intolerance to oral intake (9–11). Although delayed recovery of gastrointestinal function after abdominal surgery is not uncommon (12, 13), cases of severe DGE occurring simultaneously with intestinal paralysis after PD are still rarely reported. Compared with simple DGE, severe delayed gastric emptying combined with intestinal motility disorder may lead to longer duration of eating impairment, nutritional deficiency, and prolonged hospital stay. In addition, because the clinical manifestations of functional gastrointestinal motility disorders may be similar to mechanical obstruction, early diagnosis and treatment decisions are somewhat difficult.

**Table 1:** Definition criteria of the International Study Group of Pancreatic Surgery (ISGPS).

DGE grade	NGT required	Unable to tolerate solid oral intake by POD	Vomiting/gastric distension	Use of prokinetics
A	4–7 days or reinsertion > POD 3	7	±	±
B	8–14 days or reinsertion > POD 7	14	+	+
C	>14 days or reinsertion > POD 14	21	+	+

DGE, delayed gastric emptying; POD, postoperative day; NGT, nasogastric tube. To exclude mechanical causes of abnormal gastric emptying, the patency of either the gastrojejunostomy or the duodenojejunostomy should be confirmed by endoscopy or upper gastrointestinal radiography.

This article reports a case of severe DGE complicated by enteroparalysis after PD, focusing on its clinical manifestations, diagnostic process, treatment strategies, and long-term follow-up outcomes. Meanwhile, it aims to enhance clinicians' awareness and management of this rare postoperative complication, particularly emphasizing the long-term recovery of physical function in elderly patients and providing a reference for the management of similar patients.

## Case presentation

A 69-year-old female patient was admitted to the hospital due to “recurrent upper abdominal pain for approximately 1 month,” accompanied by symptoms of acid regurgitation, nausea, vomiting, and diarrhea. Physical examination upon admission revealed tenderness in the midupper abdomen, with no obvious signs of peritoneal irritation and no palpable abdominal mass. Her past medical history included cholecystectomy approximately 18 years ago. Admission laboratory tests indicated mild liver function abnormalities, including elevated alanine aminotransferase, aspartate aminotransferase, and gamma-glutamyl transferase. Albumin and tumor markers such as carcinoembryonic antigen, carbohydrate antigen 125 (CA125), and carbohydrate antigen 19-9 (CA19-9) showed no significant abnormalities. Contrast-enhanced abdominal computed tomography (CT) revealed an irregular mural nodule in the main pancreatic duct of the pancreatic head, accompanied by marked pancreatic duct dilation, suggestive of intraductal papillary mucinous neoplasm (IPMN). Further magnetic resonance imaging examination revealed nodular abnormal signals within the main pancreatic duct of the pancreatic head, accompanied by mild dilation of the intrahepatic and extrahepatic bile ducts, further supporting the diagnosis of IPMN.

After multidisciplinary discussion and thorough assessment of the patient's condition, pancreaticoduodenectomy (pylorus-preserving not performed) was carried out after obtaining the patient's informed consent. Intraoperatively, retrocolic pancreaticojejunostomy, choledochojejunostomy, and antecolic gastrojejunostomy with Billroth II reconstruction were completed. As the preoperative assessment suggested a likely benign nature of the lesion, no extended lymph node dissection was performed. Postoperative pathological examination revealed intraductal papillary mucinous neoplasm of the pancreas with low-grade intraepithelial neoplasia. On postoperative day 5 (POD 5), routine abdominal CT showed no significant abnormalities at the gastrojejunal anastomosis or pancreaticojejunostomy sites. A small amount of intra-abdominal fluid collection and residual gas were considered postoperative changes. However, the patient developed persistent nausea and recurrent vomiting, with symptoms significantly aggravated after eating. On postoperative day 10, upper gastrointestinal contrast examination indicated restricted passage at the gastrojejunal anastomosis (Figure 1A). After conservative treatment including gastrointestinal decompression and nutritional support, the patient's gastrointestinal function still did not recover significantly. Approximately 2 weeks after surgery, repeat upper gastrointestinal contrast examination still indicated impaired gastrointestinal passage (Figure 1B). The DGE level was grade C according to ISGPS criteria.

**Figure 1:**
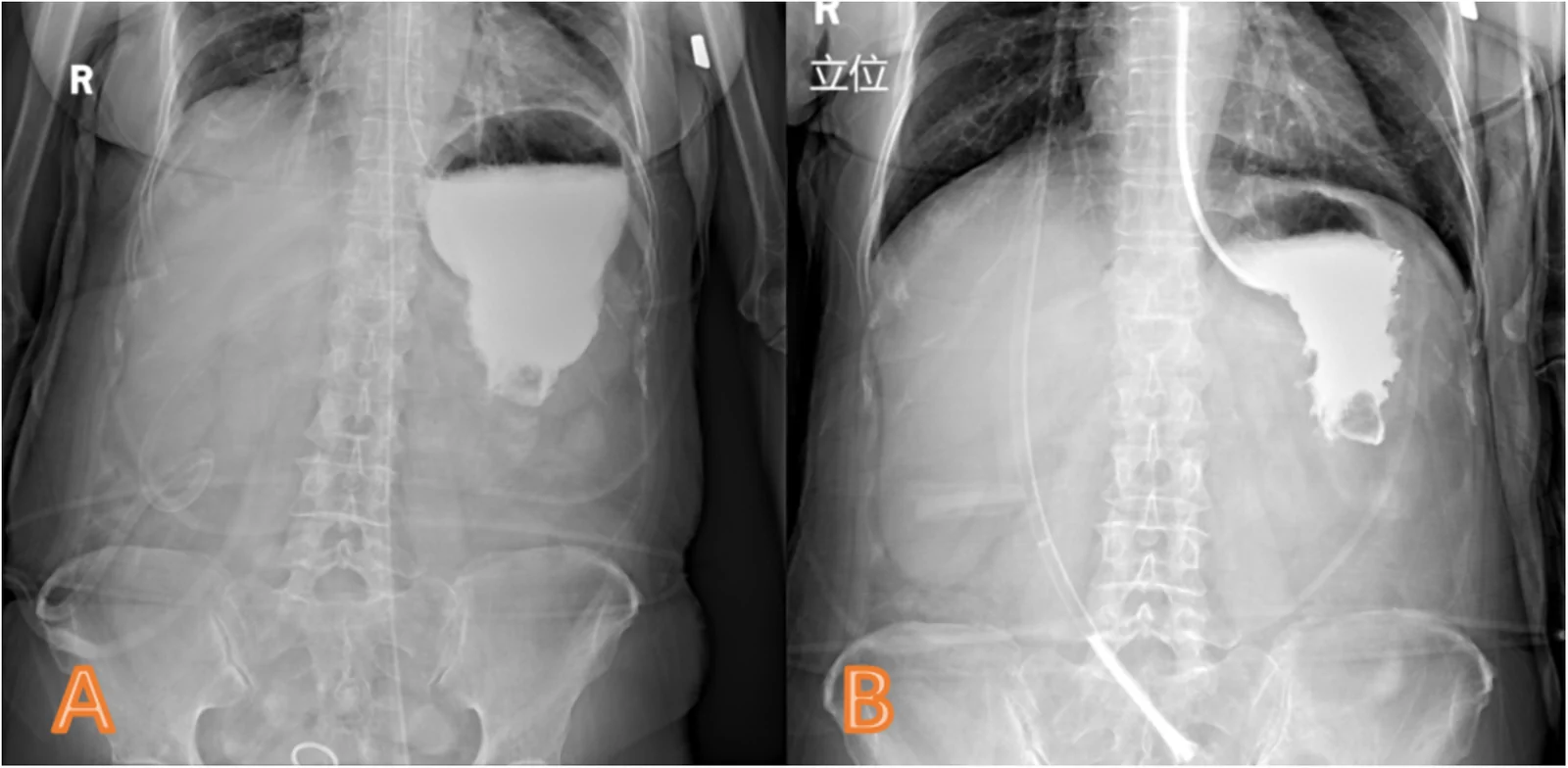
**(A)** The upper gastrointestinal contrast radiography on the 10th day after the operation and **(B)** the upper gastrointestinal contrast radiography reexamination in the second week after the operation.

Because the patient was still unable to tolerate oral intake, bowel sounds had not returned, and mechanical obstruction could not be completely ruled out, laparoscopic exploration was performed on postoperative day 25. No obvious jejunal or colonic dilation, remnant gastric dilation, anastomotic leakage, or definite mechanical obstruction or intestinal adhesions were found during the operation, but imaging could not exclude these possibilities, so Braun anastomosis was performed. After the second operation, the patient was still unable to tolerate oral intake and required continuous nutritional support. On postoperative day 31, the patient suddenly developed disturbance of consciousness and was transferred to the intensive care unit for treatment. Laboratory tests indicated electrolyte abnormalities such as hypophosphatemia and hypomagnesemia, and hypoproteinemia. A neurological examination and brain CT scan excluded intracranial diseases.

After fluid resuscitation, electrolyte correction, and supportive treatment, the patient's condition gradually improved. In order to clarify the further cause of persistent gastrointestinal dysfunction, gastroscopy was performed on postoperative day 35. The examination showed that the gastrojejunostomy anastomosis was patent, and the gastroscope could pass through smoothly, with no mechanical obstruction identified. However, significant bile reflux was observed. A jejunal feeding tube was subsequently placed for enteral nutrition support. Contrast examination via the jejunal feeding tube showed no definite mechanical intestinal obstruction, but suggested absent intestinal peristalsis; multiple intestinal dilations with gas and fluid accumulation were detected, with marked gas accumulation and dilation in the residual stomach (Figure 2A). In the following weeks, the patient received comprehensive conservative treatment, including gastrointestinal decompression, enteral and intravenous nutritional support, electrolyte management, rehabilitation therapy, and psychological comfort and counseling. Although the recovery process was relatively slow, gastrointestinal function gradually improved. On postoperative day 56, upper gastrointestinal contrast radiography showed no obvious intestinal dilation or gastric retention (Figure 2B). On postoperative day 67, the patient's gastrointestinal function recovered to the extent of tolerating oral intake, and the patient was discharged after clinical improvement.

**Figure 2:**
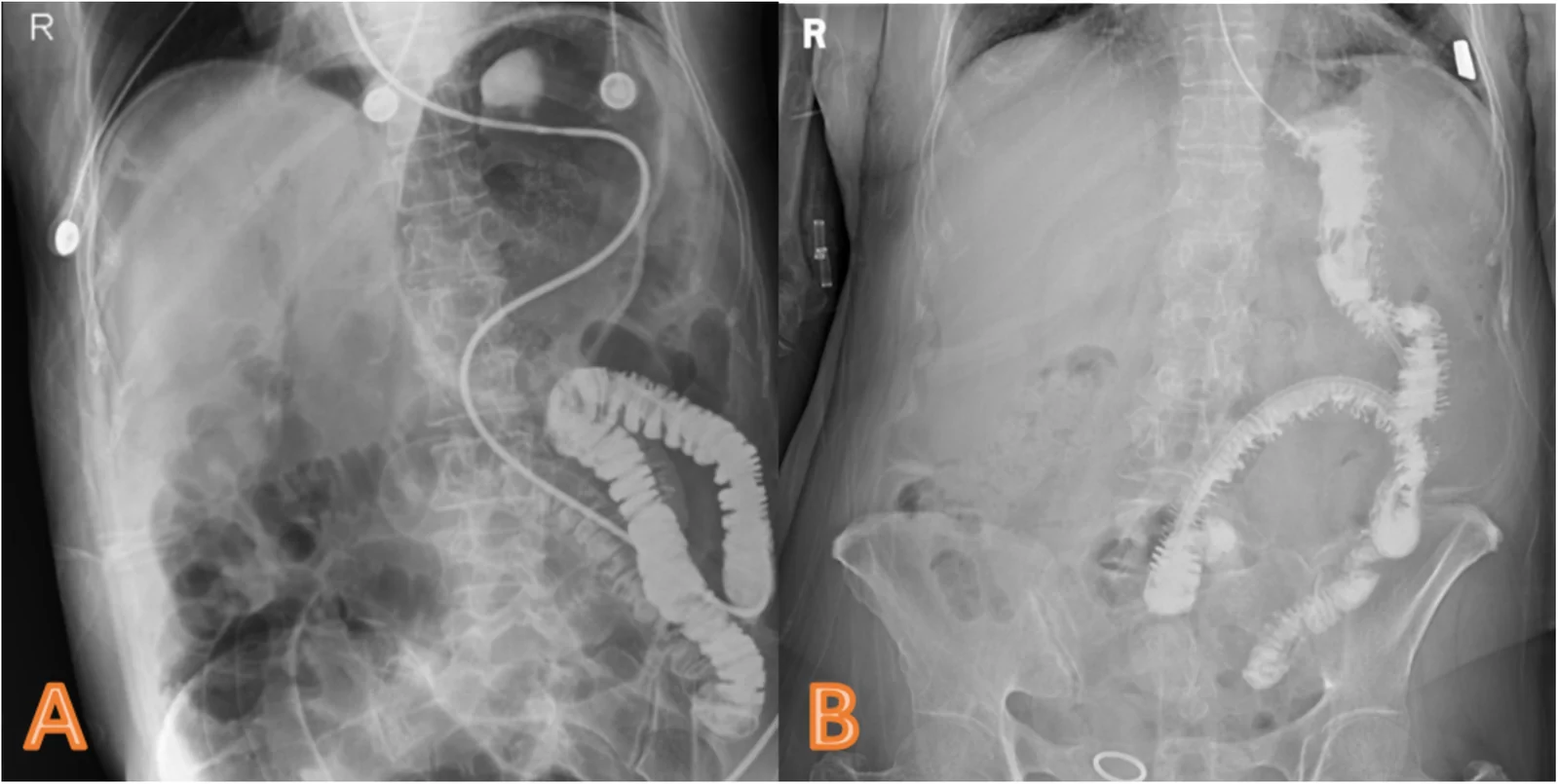
**(A)** Thirty-five days after the operation, a jejunal tube was inserted for enteral nutrition. Radiography recheck through the enteral nutrition tube showed no bowel movement and multiple intestinal dilation and obvious gas accumulation and dilation. **(B)** On the 56th day after the first operation, upper gastric intestinal tract radiography showed no intestinal dilation and gastric retention.

Long-term follow-up showed that the patient still had a certain degree of decline in physical function. About 1 month after discharge, the patient fell due to unsteady gait, and subsequently refused to walk independently for fear of falling again. At the 1-year postoperative follow-up, the patient was still unable to complete activities of daily living independently, and food intake was reduced compared with that before surgery. Later, regular rehabilitation therapy and psychological counseling were provided. At the 2-year postoperative follow-up, the patient still required family assistance with activities of daily living due to decreased mobility and physical functional limitations, but showed significant improvement compared with the early postoperative state, and was able to engage in simple social activities and independent community activities. Figure 3 shows the timeline of critical events.

**Figure 3:**
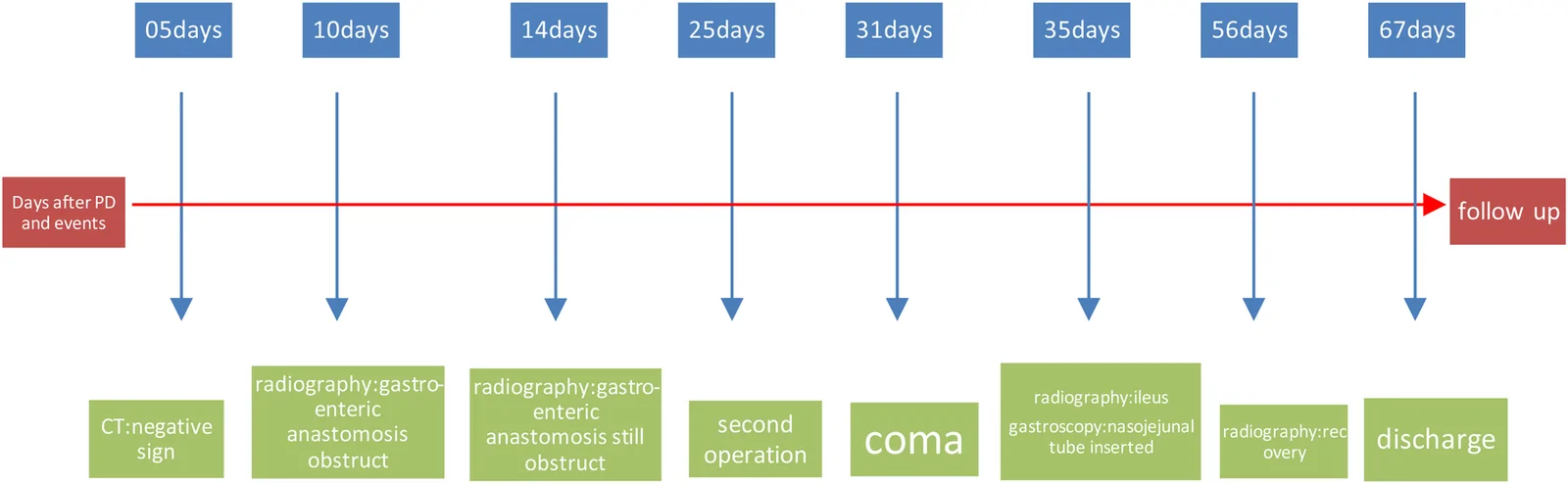
Timeline of important events.

## Discussion

PD is an important surgical approach for the treatment of diseases of the pancreatic head, periampullary region, and related conditions (1). However, due to the involvement of multiple organ resections and complex digestive tract reconstruction, the incidence of postoperative complications remains high (3, 7). DGE is one of the most common gastrointestinal complications after PD, characterized by the patient's inability to resume normal diet in the absence of mechanical obstruction, and may lead to a prolonged hospital stay, deteriorated nutritional status, and reduced quality of life (3). However, gastrointestinal dysfunction after PD is not limited to abnormal gastric emptying; persistent intestinal motility disorder (enteroparalysis), although less frequently reported, may further aggravate the clinical burden of patients. The particularity of this case lies in the simultaneous occurrence of severe DGE and enteroparalysis, suggesting that gastrointestinal motility disorder after PD may manifest as broader gastrointestinal dysfunction rather than merely delayed gastric emptying.

At present, the mechanism underlying the occurrence of DGE after PD has n't been fully elucidated, and its development may involve the combined effects of multiple factors, including surgical trauma, abnormalities in gastrointestinal neural regulation, inflammatory responses, and perioperative factors (4, 14–16). For patients who additionally present with intestinal paralysis, impaired gastric and intestinal motility may reflect a more complex state of impaired gastrointestinal motor function. Previous studies have suggested that postoperative intestinal paralysis may be associated with sympathetic nerve activation, abnormalities in intestinal neuromuscular function, electrolyte disturbances, and surgery-related inflammatory responses (4). In addition, perioperative fluid management, analgesic methods, delayed postoperative activity, and the patient's baseline nutritional status may also affect the process of gastrointestinal function recovery (6, 17, 18). Therefore, for patients with severe gastrointestinal dysfunction after PD, clinicians should not only focus on delayed the gastric emptying itself but also recognize that intestinal motility disorders may coexist and may further prolong recovery time.

When DGE and enteroparalysis occur simultaneously, their clinical manifestations are often highly similar to those of mechanical obstruction, which is one of the challenges in the diagnostic process for such patients. When postoperative patients present with persistent vomiting, gastric retention, and difficulty eating, and imaging findings suggesting restricted gastrointestinal passage, mechanical factors usually need to be ruled out first. However, early postoperative gastrointestinal dysmotility, perianastomotic edema, and delayed recovery of intestinal peristalsis can all produce imaging findings similar to obstruction. Therefore, relying on a single examination result alone may lead to diagnostic bias, and a comprehensive assessment is needed by integrating imaging, endoscopic findings, dynamic clinical changes, and the patient's overall status.

This case also suggests that for patients with persistent gastrointestinal dysfunction, a second operative intervention should be approached with greater caution. Because mechanical obstruction and functional motility disorders after PD may present with similar characteristics, in the absence of a clear anatomical abnormality, further invasive treatment may not improve the patient's symptoms and may instead cause additional trauma. Performing reoperation when input loop-related problems have not been completely ruled out may increase the patient's medical burden. Therefore, before reoperation, making full use of endoscopy and other auxiliary examinations to further clarify the etiology is of great importance. For similar cases, the treatment goal should shift from merely resolving the “passage obstruction” to clarifying the nature of the gastrointestinal motility disorder and adopting individualized strategies based on the patient's condition.

Nutritional support is a critical component in the treatment of patients with severe DGE complicated by enteroparalysis. As the recovery of gastrointestinal function may take a considerable amount of time, prolonged inability to eat can not only lead to nutritional deficiencies but may also further compromise immune function, muscle status, and overall recovery capacity. In this case, the patient was able to obtain a continuous nutritional supply even before gastric function had fully recovered by gradually establishing an enteral nutrition support pathway. Previous studies have shown that, compared with parenteral nutrition alone, the rational use of enteral nutrition may improve nutrition-related outcomes in patients after PD and promote postoperative recovery (19, 20). Therefore, for patients at risk of long-term eating impairment, gastrointestinal tolerance should be assessed as early as possible and an appropriate nutritional support plan should be established, rather than simply waiting for spontaneous recovery.

Furthermore, the long-term follow-up results of this case further suggest that recovery of gastrointestinal function does not indicate that the patient's overall recovery is complete. Although the patient eventually resumed oral intake and met the criteria for discharge, there was subsequently a decline in mobility and limitations in activities of daily living. This indicates that severe postoperative complications may exert persistent functional effects on patients, particularly elderly patients, in whom long-term nutritional insufficiency, sarcopenia, and reduced mobility may jointly affect long-term quality of life. Therefore, the evaluation of recovery after PD in patients should not focus only on short-term surgical indicators but should also include functional status and quality of life.

The concept of enhanced recovery after surgery emphasizes reducing surgical stress, promoting early mobilization, and improving overall patient recovery through multimodal perioperative management (3). For patients experiencing long-term gastrointestinal dysfunction, postoperative recovery should not end with hospital discharge, but should include continuous nutritional management, exercise training, and functional assessment. Particularly in elderly patients, establishing a long-term follow-up and rehabilitation system may help reduce postoperative functional decline and improve the ability to live independently.

Currently, there is still a lack of standardized treatment protocols for severe DGE complicated by enteroparalysis after PD. Existing treatments mainly rely on comprehensive supportive measures, including gastrointestinal decompression, nutritional support, electrolyte correction, promotion of gastrointestinal motility, and rehabilitation management. Some studies have explored the effects of chewing gum stimulation (21), optimized analgesic strategies (22, 23), and enhanced recovery pathways on the recovery of postoperative intestinal function, but the specific roles of these measures in patients with concurrent severe DGE and enteroparalysis still require further verification.

In summary, severe DGE complicated by enteroparalysis after PD is a rare but clinically significant gastrointestinal dysfunction status. This case suggests that persistent eating disorder after PD does not necessarily indicate mechanical obstruction, and the possibility of functional motility disorder should be considered. For these patients, more attention should be paid to the analysis of multifactorial mechanisms, the cautious assessment of the indications for reoperation, timely establishment of nutritional support pathways, and strengthening of long-term rehabilitation management. More studies are needed in the future to further clarify the risk factors, diagnostic criteria, and optimal treatment strategies for such patients.

## Conclusion

DGE complicated by intestinal paralysis after PD is a rare but clinically challenging postoperative complication. This case suggests that persistent gastrointestinal dysfunction after PD may present with clinical features resembling mechanical obstruction, therefore requiring comprehensive assessment integrating imaging, endoscopy, and dynamic clinical changes. For such patients, emphasis should be placed on early recognition, exclusion of mechanical obstruction, maintenance of reasonable nutritional support, and implementation of long-term rehabilitation management. Particularly in elderly patients, even if gastrointestinal function ultimately recovers, it does not necessarily mean that overall physical function has fully recovered; therefore, long-term follow-up and functional assessment are equally important.

## Data Availability

The original contributions presented in the study are included in the article/Supplementary Material, further inquiries can be directed to the corresponding author.
